# Artificial intelligence, machine learning, telemedicine, and digital transformation in nephrology and transplantation

**DOI:** 10.1080/0886022X.2025.2574537

**Published:** 2025-10-19

**Authors:** Charat Thongprayoon, Wannasit Wathanavasin, Wisit Cheungpasitporn

**Affiliations:** ^a^ Division of Nephrology and Hypertension, Department of Medicine, Mayo Clinic, Rochester, MN, USA; ^b^ Nephrology Unit, Department of Medicine, Charoenkrung Pracharak Hospital, Bangkok Metropolitan Administration, Bangkok, Thailand

**Keywords:** Artificial intelligence, machine learning, telemedicine, digital health, nephrology, kidney transplantation

## Introduction

**Figure 1.**
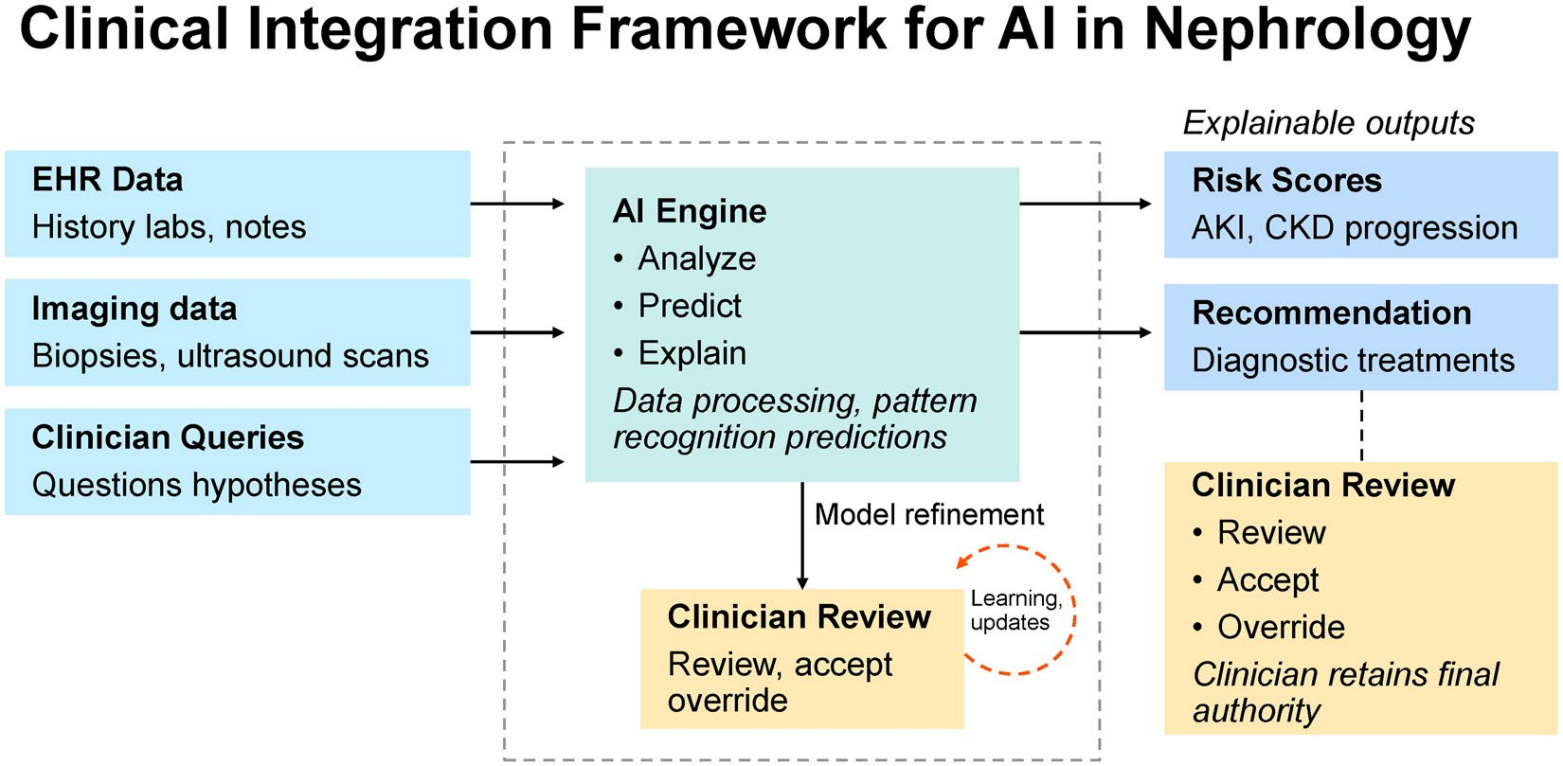
Clinical integration framework for AI in nephrology. The framework illustrates how electronic health record (EHR) data, imaging studies, and clinician queries are processed by an AI engine to generate explainable outputs, including risk scores (e.g., acute kidney injury, CKD progression) and diagnostic or treatment recommendations. Outputs undergo clinician review, with options to accept, override, or refine recommendations, ensuring that final authority remains with the clinician. Iterative feedback loops enable model refinement and learning updates, aligning AI predictions with evolving clinical practice.

The past decade has witnessed a paradigm shift in nephrology and transplantation [1,2], driven by the rapid integration of artificial intelligence (AI), machine learning (ML), chatbot (conversational agent) technologies, digital health platforms, precision medicine frameworks, and health informatics systems, including telemedicine, into research and clinical care [1,3,4]. What was once speculative is now increasingly embedded into decision-making, patient stratification, and global health strategies [1,5]. A representative example is the *Clinical Integration Framework for AI in Nephrology* (Figure 1), which illustrates how EHR data, imaging studies, and clinician queries can be processed by AI engines to generate explainable outputs such as risk scores and treatment recommendations, with final decisions safeguarded by clinician oversight. Such frameworks emphasize both the potential of AI to transform kidney care and the critical need for responsible, transparent, and clinician-centered adoption.

The *Renal Failure* Article Collection, *‘Empowering Nephrology and Transplantation: Innovations in AI, Chatbot, Telemedicine, and Machine Learning’,* represents an important milestone in this transformation, bringing together a diverse set of studies that exemplify the opportunities and challenges of digital health in kidney care.

This editorial synthesizes the contributions of this collection, contextualizes them within the broader global literature, and outlines key directions for advancing the responsible and equitable adoption of AI in nephrology and transplantation.

## Contributions of the article collection

The 11 manuscripts included in this collection reflect the breadth of AI’s applications, spanning dialysis (hemodialysis, peritoneal dialysis), transplantation, acute kidney injury (AKI), and epidemiology (Table 1) [2,6–15].

**Table 1.** Overview of articles in the renal failure article Collection on artificial intelligence, machine learning, telemedicine, and digital transformation in nephrology and transplantation.

First Author (Year)	Area of Focus	Study Design /Methodology	Key Contribution/Findings	Relevance to Clinical Practice
Zhang (2025) [7]	Hemodialysis, Symptom Management	Machine learning prediction	Predicted postdialysis fatigue in HD patients	Supports precision symptom management
Xu (2024) [10]	Peritoneal Dialysis	ML + nomogram	Predicted HF hospitalization in PD patients	Enables early intervention to reduce morbidity
Xiang (2024) [12]	Hemodialysis	External validation	Validated intradialytic hypotension model	Enhances trustworthiness of AI in dialysis
Ali (2025) [2]	Transplantation	Explainable ML	UK Live-Donor Kidney Transplant Outcome Prediction tool	Improves donor–recipient risk stratification
Li (2024) [8]	Transplantation, Fibrosis	snRNA-seq + ML + docking	Identified cell death pathways in allograft fibrosis	Advances mechanistic insights for graft survival
Thongprayoon (2023) [15]	Transplantation, Equity	Clustering analysis	Identified disparities in transplant outcomes by education	Highlights role of AI in social determinants
Gao (2024) [13]	Sepsis-associated AKI	ML using MIMIC-IV	Predicted in-hospital mortality	Aids decision-making in ICU
Lin (2024) [14]	Acute Pancreatitis + AKI	ML using MIMIC-IV	Predicted AKI risk in critically ill patients	Facilitates ICU triage and prevention
Garcia Valencia (2024) [9]	Transplant, COVID-19	Digital health + AI	Evaluated mask/vaccine recommendations for recipients	Demonstrates role of AI in infection prevention
Zhakhina (2024) [11]	Epidemiology, CKD	National database + forecasting	Modeled CKD trends in Kazakhstan	Extends AI use to population-level health
Yan (2024) [6]	IgA Nephropathy + IBD	Molecular crosstalk analysis	Novel immune links across diseases	Broadens scope of AI-enhanced discovery

*Abbreviations***:** AI: artificial intelligence; AKI: acute kidney injury; CKD: chronic kidney disease; COVID-19: coronavirus disease 2019; EHR: electronic health record; HD: hemodialysis; HF: heart failure; IBD: inflammatory bowel disease; ICU: intensive care unit; LMIC: low- and middle-income countries; ML: machine learning; MIMIC-IV: Medical Information Mart for Intensive Care IV; PD: peritoneal dialysis; snRNA-seq: single-nucleus RNA sequencing.

### AI in dialysis and symptom management

- Zhang et al. [7] applied ML to predict postdialysis fatigue, a highly prevalent yet under-addressed symptom in hemodialysis patients, offering a step toward precision symptom management.
- Xu et al. [10] developed a nomogram to predict heart failure hospitalization among peritoneal dialysis patients, highlighting the potential of predictive analytics to reduce morbidity.
- Xiang et al. [12] externally validated an intradialytic hypotension model, a critical step toward reproducible and clinically trustworthy AI tools.

### AI in transplantation

- Ali et al. [2] introduced the UK Live-Donor Kidney Transplant Outcome Prediction tool, advancing donor–recipient risk stratification with explainable ML.
- Li et al. [8] integrated single-nucleus RNA sequencing, ML, and molecular docking to delineate cell death pathways in allograft fibrosis, demonstrating how multi-omics and AI can converge to inform mechanistic insights.
- Thongprayoon et al. [15] leveraged clustering to uncover social determinants, showing distinct outcomes among transplant recipients with lower educational attainment—underscoring AI’s capacity to reveal hidden disparities.

### AI in critical illness and AKI

- Gao et al. [13] predicted mortality in sepsis-associated AKI, and Lin et al. built ML models for AKI in acute pancreatitis, both harnessing MIMIC-IV data. These studies illustrate how AI can support high-stakes decision-making in the ICU.

### Telemedicine, Chatbots, and post-COVID lessons

- Garcia Valencia et al. [9] evaluated AI-driven recommendations for mask use and vaccination among transplant recipients, reflecting the enduring relevance of digital decision support in infection prevention.

### Epidemiology and forecasting

- Zhakhina et al. [11] modeled CKD epidemiology in Kazakhstan, integrating nationwide data with forecasting tools, demonstrating the public health potential of ML beyond individual patient care.

Collectively, these works showcase methodological diversity—from predictive analytics to clustering, external validation, single-cell multi-omics integration, and digital health policy applications.

## Broader context across kidney diseases

While *Renal Failure* has been a leader in publishing pioneering AI research, the wider literature provides complementary insights:

- *Chronic Kidney Disease (CKD):* AI-driven risk equations have been developed to improve prediction of disease progression, with emphasis on transparency and interpretability [16]. These tools increasingly intersect with precision medicine approaches for individualized care.
- *Kidney Transplantation:* Deep learning models are being explored for histopathological image analysis, complementing consensus efforts in digital pathology to standardize interpretation [17].
- *Dialysis:* Machine learning–guided tools for fluid and hemodynamic management are showing promise in reducing complications and improving patient stability [18].
- *General Nephrology:* Research across high-impact journals has highlighted the importance of not just technical accuracy but also model generalizability, fairness, and patient-centered usability [1], with strong ties to the broader fields of *digital health and health informatics.*

Together, these advances underscore a shift from proof-of-concept studies toward translational applications, while real-world clinical integration remains the key challenge ahead.

## Emerging themes and insights [19–21]

1. *Clinical Decision Support in Dialysis and Transplantation*
  Predictive models are increasingly precise, but their clinical value depends on seamless integration into electronic health records and dialysis machine interfaces.
2. *Equity and Social Determinants*
  AI can illuminate hidden disparities, as shown in transplant outcomes by education level. This calls for deliberate efforts to ensure AI reduces, rather than amplifies, inequities.
3. *Multi-omics and Mechanistic Discovery*
  The fusion of single-cell sequencing with ML represents a powerful approach to unravel disease mechanisms—moving beyond prediction toward biological explanation.
4. *External Validation and Generalizability*
  The emphasis on external validation in this collection is crucial. Models without cross-cohort validation risk perpetuating ‘AI silos’ that fail in clinical translation.
5. *Telemedicine and Chatbots as Lasting Tools*

The COVID-19 era normalized digital health, and AI-driven chatbots for vaccine and mask recommendations exemplify how decision-support can extend beyond the clinic.

## Future directions

The momentum demonstrated in this collection should now be directed toward [1,2,5,22,23]:

- *Rigorous Multicenter Validation*: Moving beyond single-institution studies, leveraging multinational datasets, and harmonizing protocols.
- *Workflow Integration*: Embedding AI into clinical decision pathways and dialysis machine interfaces, ensuring usability for frontline clinicians.
- *Fairness and Ethics*: Auditing algorithms for bias, ensuring transparent reporting (TRIPOD-AI, CONSORT-AI), and engaging patients in co-design.
- *Global Inclusivity*: Expanding beyond high-income settings, as illustrated by the Kazakhstan CKD study, to ensure AI tools benefit diverse populations.
- *Regulatory and Implementation Science*: Collaborating with regulators, professional societies, and health systems to translate algorithms into approved, reimbursable, and trusted solutions.
- *Education and Capacity Building*: Training nephrologists and fellows to critically appraise AI studies, ensuring informed adoption rather than blind enthusiasm.

These priorities are summarized in Table 2, which highlights future directions, their rationale, and representative references for advancing AI, telemedicine, and digital transformation in nephrology and transplantation

**Table 2.** Future directions and research gaps in AI, telemedicine, and digital transformation in nephrology and transplantation.

Recommendation	Rationale
Rigorous multicenter validation	Most models remain single-center; external validation is essential for generalizability and clinical adoption.
Workflow integration with EHRs and dialysis machines	Predictive models must be embedded into clinical workflows and device interfaces to deliver real-time utility.
Fairness, bias auditing, and reporting standards	Transparent reporting (TRIPOD-AI, CONSORT-AI), algorithm audits, and equity considerations are needed to prevent harm.
Multi-omics mechanistic discovery	Integration of single-cell sequencing, ML, and molecular docking can reveal disease mechanisms beyond prediction.
Global inclusivity and LMIC perspectives	Most studies are from high-income settings; expanding to low- and middle-income countries ensures global relevance.
Education and capacity building	Training nephrologists and fellows in AI appraisal ensures informed adoption and sustained innovation.

*Abbreviations*. AI: artificial intelligence; CONSORT-AI: Consolidated Standards of Reporting Trials–Artificial Intelligence; EHR: electronic health record; LMIC: low- and middle-income countries; ML: machine learning; TRIPOD-AI: Transparent Reporting of a multivariable prediction model for Individual Prognosis Or Diagnosis–Artificial Intelligence.

## Conclusion

This Article Collection has highlighted how AI, machine learning, telemedicine, and chatbot technologies are already reshaping nephrology and transplantation. From predictive models for dialysis complications to mechanistic insights into graft fibrosis and public health forecasting, the breadth of applications underscores a field in rapid transformation.

Yet, the promise of AI will only be realized through validation, integration, and equitable deployment. *Renal Failure* has established itself as a hub for rigorous and forward-looking AI research in nephrology. We hope this Collection inspires future work that not only advances methodology but also meaningfully improves patient care, reduces disparities, and fosters global collaboration.

The journey is just beginning, and nephrology, perhaps more than any other specialty, is uniquely positioned to demonstrate how AI can empower medicine while preserving its central focus on the patient.

## Data Availability

No new data were generated or analyzed in this editorial.
